# The child with medical complexity

**DOI:** 10.1186/s13052-020-00935-z

**Published:** 2021-01-06

**Authors:** Manuela Gallo, Rino Agostiniani, Roberta Pintus, Vassilios Fanos

**Affiliations:** 1 Università degli Studi di Cagliari, Cagliari, Italy; 2 Area Pediatria e Neonatologia della Asl Toscana Centro (Firenze), Florence, Italy; 3 Terapia Intensiva Neonatale, AOU e Università degli Studi di Cagliari, Cagliari, Italy

**Keywords:** Children, Pediatrics, Medical complexity, Individualized medicine, Chronic condition

## Abstract

Children with medical complexity represent a big challenge for the physicians, their families and the society as well. Although there is no clear definition of this type of patients, they are affected by a chronic, often very severe condition for their whole life. They also represent a huge cost for the health care system due to their needs of continuous assistance.

In this review we summarized the definitions of child with medical complexity. Then we illustrated the strategies to treat and take care of these children in order to look at them not as a burden or a cost but as an opportunity to growth and improve as clinicians and to improve the society, to give them the best life they can live.

We also wanted to give voice to the physicians, the parents and the children themselves to really show and understand what are their experiences and their feelings in dealing with their conditions. We concluded with the description of one example of children with medical complexity: prematurity. We discussed the progresses in their treatment so far in order to illustrate what is the future of pediatrics. Since it has been more and more acknowledged that every child with medical complexity is unique, the future of pediatric is to organize an individualized approach and to “see things with the eye of a child”.

“Complex is a state of the world, complicated is a state of the mind.”

Donald A. Norman

## Introduction

The term “complexity” derives from the Latin word “complexus”, meaning something twisted, made by a multiplicity of parts which are interdependent on one another. A situation can be considered complex because it originates from the intertwining of different elements that interact with each other, creating disorder and causing uncertainty. In a complex situation, it is difficult to identify and manage all the variables involved, just as much as it is essentially impossible to predict its progresses. A problem that we define complex does not present a univocal solution, but it needs to be globally considered, analyzing all the elements that compose it and their interactions.

Complex and complicated: often in managerial language these two concepts are used interchangeably. One speaks about a “complex situation” or a “complicated problem”, using the two concepts as synonyms. But they are not.

The term “complication” derives from the Latin “cum plica” from which “complicare”, and it indicates something bent, wrapped around itself. A problem is complicated when it shows itself as a result of a combination of parts difficult to codify. Untangling the complication can be exhausting, but there is a solution anyway. What is complicated can be reduced to something simpler.

We found it necessary to premise these definitions to highlight the objective difficulty in managing the child with medical complexity. In English literature they speak about “complex chronic” or “medically complex” as well.

We report as an example among the many cases of child with medical complexity, that concerning a 10 years old child affected by CLOVES Syndrome (*Congenital Lipomatous Overgrowth, Vascular malformations, and Epidermal nevi):* during his life he has undergone 16 major surgical procedures, 40 NMR, needed 14 different types of Medical Specialist and he was forced to deal with many travels with his family.

### How big the problem is?

In the last years, the epidemiological landscape of the pediatrics diseases has changed: we are facing a progressive increase of the number of children suffering from chronic diseases (*children with special health care needs* [CSHCN]), mainly due to improvement in the level of health cares and to the subsequent increase of the survival rate. Among the CSHCN, those with medical complexity (CMC) are, without a doubt, the most fragile from the medical point of view and with the most intensive healthcare needs. The management of these patients needs specific skills and new strategies that should involve the pediatricians in the hospital as much as in the homecare [1].

In the US, about 3 out of 76 millions of children shows a medical complexity such that the category of CMC is increasing at a rate of about 5% per year, exceeding the growth rate of the children as a whole [1].

In particular, it has gone from 3.000.000 cases in 2013 to over 3.600.000 in 2017.

In Italy, for example, the percentages of hospitalization in 2012 at the Meyer Pediatric Hospital in Florence, are represented for the 35.8% by children with congenital malformation, and for the 22.7% by children with neurodevelopmental disorders.

### Who are the children with medical complexity?

The Maternal and Child Health Bureau (MCHB) defined the CMC those who are affected by or are at risk of developing a pathology that can alters the neurological development, chronic behavioral or emotional problems and who need diversified and more intense health care, compared to that needed by the general pediatric population [2].

There is still no clear definition of CMC, nevertheless, to detect this kind of patients, in the English literature, the following features are acknowledged and increasingly accepted [3, 4]:

1) chronic and severe health conditions;

2) substantial need of the National Health Care System;

3) functional limitation, often severe;

4) high utilization of health resources.

It should be noted that in Italy, the first three above-mentioned characteristics are acknowledged for the detection of CMC, while the fourth cardinal point is represented by the “rare frequency” of this condition.

More technically, the *Children’s Hospital Association,* defines the CMC in groups of clinical risk (CRG) of 5b degree or higher (as shown in Table 1), including the following categories: Single Dominant Chronic diseases, Complex Chronic diseases and Neoplastic diseases [5].

**Table 1:** Classification of the *Children’s Hospital Association*

	***NON-CHRONIC*** ***(CRG 1,2)***	***EPISODIC*** ***CHRONIC*** ***(CRG 3,4)***	***LIFELONG CHRONIC*** ***(CRG 5a,5b)***	***COMPLEX CHRONIC*** ***(CRG 6,7,9)***	***MALIGNANCIES*** ***(CRG 8)***
**Description**	- Acute pathological condition lasting less than one year	- Pathological condition lasting more than one year that manifests itself in an episodic manner; - potentially treatable with adequate treatment; - likely not to manifest in adulthood	- Severe primary pathological condition, involving an entire apparatus and more likely to cause long-term damage	- Severe chronic pathological condition involving two or more apparatuses or - chronic pathological condition with progressive deterioration or - technology-dependent pathological condition	- Highly malignancy pathological condition that requires timely treatment
**Examples**	- Head trauma, peritonitis, UTI	- Asthma, major depression	- Congenital heart disease, short bowel syndrome	-Cerebral palsy, intellectual disability	- Neuroblastoma, acute myeloid leukaemia

Taken from “*Children and Young Adults with Medical Complexity: Serving an Emerging Population”, Edwin F. Simpser* et al. Modified [5].

The most representative diagnoses include: chronic lung diseases, genetic diseases, prematurity and its complications, congenital cardiac disturbances, cerebral palsy, neurological pathologies, orthopedic and respiratory diseases, HIV/AIDS, eating or metabolic disorders, spina bifida, head trauma, sickle cell disease, short bowel syndrome and congenital anatomical malformations. These diagnoses are often accompanied by one or more comorbidities, such as developmental delay and intellectual disability. The disability could be permanent or temporary and affects without a doubt the life of the children such that they cannot take part in the normal activities that characterize the different phases of their development. Successively, among all these examples, we will analyze two problems among those above-mentioned: prematurity and intellectual disability. Furthermore, we will discuss the role of palliative care for these children.

The daily assistance of the CMC is, thus, very problematic and it develops on different levels, among which there are 3 that are fundamental: drugs administration, respiratory and nutritional support. The security risks addressed by the CMC are, for the most part, due to the complex nature of their care rather than the specific risks posed by the complicated nature of their pathologies.

It must be strongly emphasized that, precisely by virtue of these characteristics, these children highlight the vulnerabilities of our approach to the cures: fragmented and often inefficient [6].

Furthermore, although CMC represent < 1% of the pediatric population, they use almost a third of all pediatric expenses for the health care [7].

Data from US show that the most important items of healthcare expenditure due to CMC are represented by the neurologic/neurosurgical diseases for the 30%, by hematologic pathologic conditions for the 13%, by neonatal intensive care for more than the 10% but, even more interesting data, is that 1 out 4 hospitalization of these little patients could (and should) be avoided [8].

Thus, a clear and shared strategy is necessary for the management of CMC, the aims of which should be: improve the experience of these children in the healthcare facilities and the quality of the cares, improve the results (for the children themselves and for their families) and reduce the future healthcare costs [9].

In this sense, the “NACHRI”, (National Association of Children’s Hospitals and Related Institutions) recently elaborated a program that develops on three levels:

1. Create a national database to measure the assistance of CMC, relative costs and share information
2. Create a regional network to share clinical data with the different health care professionals who assist these little patients
3. Create domestic care services to mitigate the severity of child’s chronic conditions

Thus, the beginning of the care of CMC must be precocious, global and extended to the family, the management must be multidisciplinary (from the interaction to the integration) with a multi-specialized staff and a pediatric coordination center and the network of care must include the birth centers, the centers of references, the family pediatrician and the territorial sanitary garrisons [10].

### The experience of the children with medical complexity, of their parents, of the health care professionals, of the school and the society

#### The health care professionals

Often, the reactions of health care professionals to the CMC tend to be elusive: “I do not have time to deal with all this”, “these pathologies are unfamiliar to me”, “I am not paid to do all this”. Therefore, the CMC switches from the management by the pediatrician from that by the specialist. It is very important that the health care professionals think about their behaviors towards the patients, in particular those with chronic diseases, and even more, the little patients with medical complexity.

A recent study showed, interestingly, the significant change of the health care professionals after a training course, with regard to the problems that we are illustrating (see Table 2) [11].

**Table 2:** Characteristics that identify the perception of the child with medical complexity, before and after a training course

***Before the training***	***After the training***
Chronic disease	Chronic condition
Multiple problems and drugs	Need for support for daily activity
Home technology	Technology addition
Follow up	Frequent appointments and hospitalizations
Multidisciplinary approach	Team approach

Taken from [11]. Modified

#### The parents

A scientific paper of 2019*,* revealed that the parents of these little patients have a sense of great awareness of the difficulties, from the health care professionals, to take care of their children, but of the importance of their role as well [12]. In this regard, we report three brief observations made by as many parents.

*“They are the professionals, we are the professionals of our children.”*

Many parents perceive the scarce collaboration from the members of the staff and are suffering for the lack of communication and interaction among the different medical specialists.

*“A doctor often does not ask another doctor.”*

Furthermore, the parents are open to “negotiate” the boundaries and the limits of their assistance.

*“When a nurse, see that you (parent), do the right thing at the right moment, they are more inclined to let you do (it).”*

Thus, the collaboration between the health care professionals and the parents is essential, in particular in case of CMC, whose parents are specialized health care professionals themselves. In addition to the reinforced partnerships with the health care professionals, the needs expressed by the parents include a better communication with the staff and a greater attention to the continuity of care. In fact, the participation of the families in the cures and the assistance of their own child, represents one of the most significant indicators of quality of the Healthcare System.

#### The school

The school plays a fundamental role in the difficult growth path of the CMC and in supporting their families.

In this respect, the CAPHC (*Canadian Association of Paediatric Health Centers*), has done an excellent job in April 2018, creating some Guidelines concerning the management of these little patients, including the school environment as well [13].

In Italy, the scholastic integration of the students with physical and intellectual disability constitutes a strength point of our educational system, aiming at the creation of a welcoming community in which all students, despite their functional diversity, can experience individual and social growth. Full inclusion of the students with disabilities is a goal that the school pursues through an intense and articulate planning, valorizing the internal professionalisms and the resources offered by the territory. The MIUR implements several support measures to favor the integration: support teachers, funding of projects and activities for the integration, training initiatives for support curricular teachers and the administrative, technical and auxiliary staff [14].

However, it should be stressed that, even in more developed countries, the care of CMC is too often “reactive” rather than “preventive” or “anticipatory”, therefore the path for an effective and holistic assistance is to date still very long.

#### Society and politics

A detailed examination of this aspect goes beyond our objectives.

Nevertheless, it is interesting to note that some States have made considerable progresses in attempting to guarantee an adequate assistance to CMC. For example, the American society has taken a huge step forward in 2019 by making law the ACE Kids Act *(Advancing Care for Exceptional Kids*) [15].

The ACE Kids Act supports a better coordination of the CMC cares, the home assistance and in the different places of the community, reduces the unnecessary admissions and provides an easier access to care beyond the state borders, when needed.

#### The patients

It is in no way possible to describe in an objective and timely manner the life of a CMC, their emotions and their ambitions. Let us therefore leave room for the words of a patient, an American young adolescent, who admirably synthesize everything you need to know:

“Since the team that takes care of me, taught me the abilities that I needed, I am independent and I will be able to go on with my adult life with the necessary supports. I will soon be an adult with medical complexity and the way to self-manage, that I learned, will allow me to satisfy my needs and reach my goals” L. Rodgers.

### One example of children with medical complexity

#### The preterm newborn

Let us consider the prematurity and its complications. In the last 10 years the percentage of prematurity has remained virtually unchanged (1 out of 10 new births), while the percentage of very preterm newborns that are part of CMC, has increased. This increment is strictly related to, on one hand to the increase of the artificial fertilization techniques, and on the other hand, to the improvement of the quality of the care that allows for a reduction of the perinatal mortality even in case of very low gestational age and greater outcomes in the long term as well.

There is a very spirited debate concerning the “dawn of life” in the literature: *Do we really want what we want?* [16].

It is very difficult to objectively define the limit of the ability to survive: scientific criteria are formulated in very imprecise terms and they depend on the available technological resources.

In Japan, for instance, they are investing many resources to improve the survival rate of the newborns born between the 22 and 23 weeks of gestation, although the knowledge and the medical technologies are limited for the care of these neonates and on the long-term outcomes [17].

Several studies have suggested that the therapies for the treatment of the preterm infant may have consequences in adult life. For instance, it is known that until the early 1990s, the majority of preterm newborns, were extensively treated with corticosteroids to prevent or reduce the incidence and severity of chronic lung disease [18]. In fact, corticosteroids improve respiratory function and reduce the need for both mechanical ventilation and oxygen administration, resulting in a decreased mortality. However, recent studies have shown that the treatment with corticosteroids may also have negative long-term effects on the heart, possibly initiated by inhibiting the cardiomyocyte mitosis, and resulting in a reduced number of cardiac muscle cells in adulthood. These changes appear to elicit a late systolic dysfunction, due to compensatory cardiac dilation [19].

Moreover, preterm infants, given their low nephrons number, are considered at a higher risk of congenital oligonephronia, as well as low birth weight neonates, linking body weight at birth with the susceptibility to develop CKD in adulthood [20].

Many studies have shown that prematurity is a risk factor for the development of neurological diseases, such as Parkinson’s and Alzheimer’s disease in later life [21].

Cortical development is a complex process that follows a well-defined chronology: neurogenesis and neuronal migration continue until the end of the second trimester while synaptogenesis, brain folding, and myelination are in progress after premature birth. Consequently, preterm newborns are predisposed to disruptions in cortical connectivity, cell death, and myelination disorders. The negative effects of these processes may clinically appear only after a long time, during adolescence or in adult life [22].

These and many other examples make us understand that we need to pay close attention to CMCs. Diseases that manifest during the early stages of life, such as happens in preterm babies, can also have important consequences on their future as adults. In fact, complications of prematurity may influence the quality of life, with serious long-term consequences in adulthood.

### Pediatric palliative care

Life-limiting conditions (LLCs) in children have been defined as conditions for which there is no reasonable hope of cure and from which children will die. Life-threatening conditions are those for which curative treatment may be feasible but can fail, such as cancer [23].

Palliative care for children and young people (PPC) with life-limiting or life-threatening conditions is an active and total approach to care, from the point of diagnosis throughout the child’s life and death. It embraces physical, emotional, social, and spiritual elements, and focuses on enhancing quality of life for the child/young person and supporting their family [24].

According to the Association for Children’s Palliative Care (ACT) and the Royal College of Paediatrics and Child Health (RCPCH), we can identify four categories of children eligible for PPC:

- category 1: children with life-threatening conditions for which curative treatment may be possible, but can fail (for example cancer, organ failure of heart, liver or kidney, infections);
- category 2: children with conditions requiring long periods of intensive treatment aimed atprolonging life and allowing participation in normal activities, but where premature death is still possible (for example cystic fibrosis, HIV/AIDS, cardiovascular anomalies, extreme prematurity);
- category 3: children with progressive conditions without curative options, where treatment is exclusively palliative and may commonly extend over many years (for example neuromuscular or neurodegenerative disorders, progressive metabolic disorders, chromosomal abnormalities, advanced metastatic cancer on first presentation);
- category 4: children with irreversible, not progressive conditions with severe disability causing extreme vulnerability to health complications (for example severe cerebral palsy, genetic disorders, congenital malformations, prematurity, brain or spinal cord injury) [25].

Medical and technological progress allow newborns, children and teenagers suffering from lifelimiting and life-threatening illnesses to survive, but not necessarily to recover. Therefore, in recent years, the number of children needing PPC has been steadily increasing.

Several scientific studies have shown this.

A British study estimates that more than 40,000 children (0–19 years) in England in 2009/2010 were living with an LLC [23]. A more recent study shows that these children are more than 86,000 in 2017/2018 [26]. The highest requirement of children with LLCs for palliative care occurs in the first year of life and decreases during childhood [23, 26].

In Italy the numbers of patients involved in hospital stays for LLC and life-threatening diseases eligible for PPC were 35,734 in 2001, 37,040 in 2002 and 38,641 in 2003. For the patients with non-oncological diseases, the maximum rate occurred in children aged 0 to 2 years, with a peak in the first month of life [27].

The need for PPC globally is unknown. Making accurate estimates is important to understand the scope of the need and to advocate to meet it [28].

These children can be considered part of CMC.

The main elements of children’s palliative care services include: medical care, 24 h nursing care, social care, education, play, therapies, short breaks, leisure and emotional support. There needs to be co-ordination of these services to enable seamless and holistic support for families.

Global institutions and interested people in children’s health and well-being should be more interested in and make PPC accessible to all children who need it.

## Conclusions

In this review we have outlined some fixed points in the definition, in the approach and in the experience of CMC and in the implications of this complexity for families, health professionals and society.

The Pediatrics of the future is more and more technological and advanced but “in medicine, the high tech, meaning the super-technology, cannot and must not make us forget the high touch, meaning the human connection” (J. Naisbitt, High Tech/High Touch) [29].

The goal of the Pediatrician is to take care of the children, but also to communicate with them and the family and establish a therapeutic alliance and, not least, “see things with the eyes of the child” [30].

This is all the more true the more complex the pathology.

We cannot fail to mention, in conclusion, a famous phrase by Singer:

“Every human being has the right to the highest quality of life he can achieve”.

(Sanctity of life or quality of life? Singer, Pediatrics 1983)

## Data Availability

“Data sharing is not applicable to this article as no datasets were generated or analysed during the current study.

## Abbreviations

CLOVES: Congenital Lipomatous Overgrowth Vascular malformation and Epidermal nevi
CSHCN: Children with Special Health Care Needs
CMC: Children with Medical Complexity
MCHB: Maternal and Child Health Bureau
CRG: Clinical Risk Group
NACHRI: National Association of Children’s Hospital and Related Institution
CAPHC: Canadian Association of Pediatric Health Centers
MIUR: Ministero dell’Università e della Ricerca meaning Ministry of education, university and research
ACE: Advancing Care for Expectional kids
LLCs: Life-limiting conditions
PPC: Pediatric Palliative care
ACT: Association for Children’s Palliative Care
RCPCH: Royal College of Paediatrics and Child Health
